# Considerations on the Treatment of Typhoid Fever

**Published:** 1847-05

**Authors:** 


*Considerations on the Treatment of Typhoid Fever.*—By M. GENDRIN.

Typhoid Fever,—the dothinenteritis of Bretonneau, and typhus abdominals of the Germans, has lately become unusually common in Edinburgh. It may be said to be the prevailing form of fever in both that city and in Glasgow, a circumstance sufficiently curious, when it is remembered that, for several years past, no intestinal lesion had accompanied the disease. It was first remarked by Prof. John Reid, when pathologist to the Edinburgh infirmary, that the few cases which appeared there, always came from the country, more especially from Linlithgow, or Fife, and never originated in the city. Dr. Hughes Bennett, the present pathologist, has for more than three years, had occasion to confirm the truth of this observation, and lately stated (see report of Ed. Medico-Chir. Society, Month. Journ., June, 1846,) that during that time, only two cases had occurred in the infirmary. During the present winter, the disease has been very common, and he has examined upwards of twenty cases, in which the follicular lesion of the intestines has been more or less well marked. Under these circumstances, observations on the treatment of this affection, by so experienced a physicion as M. Gendrin, condensed from a lecture he lately delivered, may be considered as particularly well timed.

According to M. Gendrin, typhoid fever must inevitably pass through its three stages of increase, intensity and decline. He insists on this, because the practical rules which result from it, are, that the disease cannot be cut short, that the expectant system is the basis of our treatment, and that the active interference of art ought to be limited to accelerating the evolution of the disease, and to moderating the accidents which arise during its course.

Thus, in a slight case of typhoid fever, M. Gendrin only follows the expectant system of medicine. He causes it to consist in surrounding the patients with all the hygienic precautions compatible with their social position; submitting them to a dietetic regimen, having relation to the state of their digestive organs, and removing all those moral influences which may act injuriously upon their minds or bodies. In this manner a cure is effected in a considerable number of cases.

When the disease is severe, a more active treatment is necessary, consisting of blood-letting, evacuants, baths, epispastics, alteratives, &c.

M. Gendrin believes it useful to practice one or two bleedings from the arm in the first stage; but when this has passed, when the petechial eruption is developed, he considers no benefit is to be thus obtained. Local blood letting, according to him, causes more injury than benefit, for leeches or cupping applied to the abdomen, act at too great a distance from the inflamed intestine, to render them efficacious; and, on the other hand, a quantity of arterial blood is removed, which enfeebles the patient without doing any good.

In many cases, dyspeptic symptoms may be observed at the commencement, or during the progress of the first stage. All the individuals who enter his wards with stomach symptoms take an emetic with the best results. Typhoid fevers, accompanied at their commencement with symptoms of prostration, are transformed into a benign fever, under the influence of a vomit. Such was the treatment the French found to succeed completely at Vienna in 1809. It is only necessary that the emetic should be administered at an early period, for when the petechial eruption has occurred, and ulcerations have taken place in the small intestines, or an erythematous state in the stomach, the remedy is more hurtful than beneficial

M. Gendrin strongly condemns the indiscriminate use of purgatives, more especially drastic ones, which he rejects altogether.—The only agents of this class he employs are gentle aperients, emeto-cathartics, which, in addition to a local, conjoin a general action. He prescribes these remedies at the commencement, and sometimes during the decline of the disease, when there remains some flatulence, and liquid matters remain in the intestines, as is announced by gurgling. A saline purgative will then excite the action of the intestines, and decide the convalescence.

M. Gendrin has employed cold effusions in typhoid fever for fifteen years, causing water to trickle over the surface, or to be applied with a sponge. He considered it most efficacious in moderating the symptoms of the first stage, and it is very grateful to the patient. The subtraction of caloric in this way causes the pulse to fall from 120 to 80 and even to sixty beats in the minute. It sometimes happens that the pulse very slowly rises again, and therefore it is necessary to proportion the subtraction of caloric to the reactive powers of the individual. It should not be applied in the first stage, for after the eruption, reaction is not possible.

Tepid baths, on the other hand, are applicable exclusively to the adynamic period. They then serve to re-establish the action of the skin. It is necessary to prevent their too prolonged use, and too high temperature, otherwise they become debilitating.

Epispastics, more especially blisters, are at present very little employed.

In the last stage tonics are often necessary, especially combined with nutritive substance. M. Gendrin gives, with beef tea (bouillon,) the wine of quinine ; which has the advantage of uniting a fixed tonic with a diffusible stimulus. Wine, associated with bitter and aromatic substances, he prefers to camphor, which is only useful when there are ataxic symptoms.

Lastly, M. Gendrin speaks of the medicines which act chemically. They are, Seltzer water, chloride of sodium, alkalies, and chlorine, administered internally. All these preparations, according to the Professor of la Pitie, have no great advantages to reccommend them. The effervescing lemonade only constitutes an agreeable drink, the action of which, slightly stimulating, may be rendered serviceable. With regard to the chlorates and chlorurets, they have such an insupportable odour, as to be rejected for this cause alone, even were their therapeutic inutility not proved by experience.—*Monthly Journal of Medical Science, from Journal de Medicine et de Chirurgie Pratiques.*

